# Assessing Preferences for Animals in Children with Autism: A New Use for Video-Based Preference Assessment

**DOI:** 10.3389/fvets.2017.00029

**Published:** 2017-03-10

**Authors:** Noémie A. Guérin, Kerri E. Rodriguez, Matthew T. Brodhead, Marguerite E. O’Haire

**Affiliations:** ^1^Center for the Human-Animal Bond, Department of Comparative Pathobiology, College of Veterinary Medicine, Purdue University, West Lafayette, IN, USA; ^2^Department of Counseling, Educational Psychology, and Special Education, Michigan State University, East Lansing, MI, USA

**Keywords:** preference assessment, reinforcer assessment, animal-assisted intervention, autism spectrum disorder, applied behavior analysis

## Abstract

The inclusion of animals into interventions for children with autism spectrum disorder (ASD) is a growing practice known as animal-assisted intervention (AAI). The choice of the animal to include in an intervention is often solely up to the interventionist and depends on their experience, subjective judgment, and ease of access to different animals. For individuals with ASD who are non-verbal and unable to indicate preferred stimuli or activities, incorporating preference into interventions has been linked to increases in positive behaviors and enhanced quality of life. We propose that animal choice based on a participant’s preference may enhance the experience of AAI and maximize its outcomes. A common technique used to reliably determine preferred interactions and activities in interventions for children with ASD is a stimulus preference assessment. The video-based multiple-stimulus without replacement (MSWO) procedure, in particular, allows for discrimination of complex stimuli that could not feasibly be presented all at once, which is the case when choosing an animal. Based on the well-documented reliability of this technique in the field of applied behavior analysis, we propose that a future direction in AAI is utilizing video-based MSWO to guide animal selection.

Animals are increasingly included in interventions targeting children with neurodevelopmental disorders, including autism spectrum disorder (ASD) (1). ASD is characterized by a deficit in social communication and restricted, repetitive patterns of behaviors and interests starting in the early developmental period (2). These symptoms result in social impairments, amplified emotional responses, and stress for the child and his/her family (3). The use of animals to target these difficulties, known as animal-assisted intervention (AAI), is a popular practice and is at the center of a growing body of research (4). This popularity is further evidenced by the finding that an estimated one in four children with ASD has participated in some form of AAI (5).

The umbrella term AAI comprises any intervention using animals in support of human health or well-being, including animal-assisted therapy (AAT), animal-assisted activity (AAA), and animal-assisted education [AAE (6)]. During AAT, an animal is specifically incorporated into therapy with a trained interventionist such as a social worker, counselor, or medical professional (7). In contrast, AAAs do not feature a structured intervention or a predetermined therapeutic outcome but instead occur in situations in which an animal is partnered with motivational or recreational activities (7). Finally, AAE refers to the inclusion of an animal in an educational setting. This broad definition of AAI encompasses a number of practices with varied research methodologies, target populations, and outcomes reported in the scientific literature (4). Common types of AAI for children with ASD include animal visitations to treatment centers (8), literacy programs (9), therapeutic horseback riding (10), and the inclusion of pets in the classroom (11).

The rationale supporting the inclusion of animals in services for with children with ASD is that an animal may provide a non-judgmental, soothing presence (12) that could calm or bring emotional stability to a child (13). Animals have also been reported to increase social interactions and act as a transitional object, and thus may facilitate communication between a child with ASD and his or her therapist, peers, or family (14–16). Studies have also found that the presence of an animal during therapy can decrease problem behaviors such as physical and verbal aggression (17), while increasing positive emotional expression (15, 17, 18). These findings extend to the school setting, where interacting with guinea pigs has been reported to increase social interactions (19) and reduce physiological arousal (20) in children with ASD.

A variety of animal species can be included in AAI, though domestic species are often recommended to both ensure the safety of the participants and maximize welfare for the animal. The most common species included are dogs, horses, small mammals (e.g., guinea pigs, rabbits), and domestic farm animals [e.g., dairy cows, sheep (4)]. Because of the availability of multiple species of animals, the selection of the animal to include in AAI is generally an open choice for the interventionist. Sometimes, specific therapeutic goals such as physical/motor skill development can inform the choice of the animal. For example, equine-assisted therapy is indicated to improve motor functioning, as the rocking movement of the horse’s gait helps relax the lower body (21). Other times, the animal is included in an intervention for the larger aim of providing social support or facilitation, roles that can be held by a number of different animal species (22, 23). In these cases, the choice of the animal may rely on availability or simple convenience rather than based on individual need. For example, an interventionist may choose to include the animal that is the most accessible to them—typically their own pet—rather than an animal of a specific species.

The attitude of children toward animals varies from liking certain species to having a phobia of others and is significantly associated with their age, sex, ethnicity, background, and most importantly their personal experience (24). Even though children with ASD have been reported to have a lower rate of animal phobias than chronologically age-matched controls, those who do have animal phobias are more likely to show problem behaviors (25, 26). For these children, selecting the right animal can make the difference between presenting a source of enrichment or a trigger for panic.

Research on the incorporation of individual preference in populations with neurodevelopmental disorders such as ASD has grown substantially in past decades and has been shown to be an important consideration for intervention success (27). While typically functioning individuals are easily able to verbally indicate their preferences, low-functioning individuals with ASD are often unable to effectively communicate what they like and dislike due to core deficits of ASD (2). A solution to help mitigate this deficit in communication and increase the quality of life of these individuals is the use of a preference assessment, a technique that has been shown to reliably assess preferences without the need for verbal communication [see Ref. (27) for review].

Behavioral studies have found that incorporating choice into the daily lives of individuals with severe developmental disabilities can result in an increase in appropriate positive behaviors, decreases in problem or challenging behaviors, and enhanced task engagement and participation (28–30). Further, incorporating choice into areas such as vocational and job selection have now allowed those who cannot express their preferences verbally to experience improved job performance, increased job satisfaction, and enhanced quality of life (31–33). Behavioral researchers have also demonstrated that opportunities to make choices can function as a reinforcer (34). Therefore, along with the benefit of identifying preferred stimuli, choice-making has the ancillary benefits of enriching lives of individuals with ASD.

Based on the encouraging findings of behavioral research on choice and preference, we propose that incorporating choice into AAI has the potential to improve positive outcomes and engagement while reducing problem behavior by incorporating the participant’s desires and/or aversions into animal selection. By incorporating the widely used stimulus preference assessment (SPA) tool into the practice of AAI, low-functioning individuals can voice their own opinion prior to an animal interaction. Evaluating preference for an animal can increase the probability that a preferred animal is selected and individual autonomy respected. Furthermore, preference assessments may also help to rule out possible animal phobias that could increase the efficacy of AAI by avoiding discomfort and maximizing any positive outcomes to be gained. In this paper, we discuss the potential of SPAs for use in AAI, particularly to inform animal selection while offering suggestions and guidelines to both researchers and clinicians.

## Overview of Stimulus Preference Assessments

Preference assessments first emerged in applied behavior analysis (ABA) research as a way to identify effective reinforcers in participants with low verbal communication. In one of the first attempts to assess preferences in non-verbal individuals, Pace et al. (35) designed a study in which items were successively presented in front of an individual. Those that were chosen or approached more often were assumed to have a higher reinforcing value for the individual than those that were either not chosen or chosen less often (35). The reinforcing value of these items could then be verified through an experimental evaluation (36). In recent literature, preference assessments are most often used to identify individualized reinforcing stimuli to offer as a reinforcer during a behavioral intervention. These assessments are methodologically rigorous and have been shown to be more reliable and effective at assessing preference and identifying reinforcers (37) than the sole reliance on parent and caregiver suggestions (38, 39), making them a potentially valuable asset to the field of AAI.

The structure and format of preference assessments have considerably varied over time (37). Early approaches used touching an item as an indicator of preference, with only one stimulus presented at a time (35). Later, a forced choice format between stimuli was found to be a more accurate predictor of subsequent reinforcing value, first with two stimuli (40), then with multiple stimuli (41, 42). The multiple stimuli method, in particular, has shown strong predictive validity for identifying verified reinforcers and is markedly more efficient than other methods (37, 43).

Whether in a single, paired, or multiple stimuli format, preference assessments have previously been limited by size and modality; that is, in order to be presented on a surface the choice items must be both small and physically available. While this is practical with toys and other tangible items such as food, this method is logistically challenging when desired stimuli are either not present at the time of assessment, too large to be presented in this format (e.g., a playground), or represent a leisurely activity which cannot be represented by a single item (e.g., taking a walk outside). With the development of evaluation methods that are increasingly efficient and sensitive to practitioner needs, researchers have addressed this limitation by presenting non-tangible and non-accessible stimuli with alternative formats such as printed words, static pictures, and most recently with dynamic video formats (27).

Video technology has emerged as a particularly useful tool for interacting with individuals with severe disabilities (44, 45). As a method of stimulus presentation, videos provide more contextual information than pictures or words, and can increase the salience of the stimuli. Video-based preference assessments have been validated as a comparable measure to tangible preference assessments, with studies showing reliability of choice across those two methods (46, 47). Thus, a video-based SPA is appropriate for choices between complex stimuli such as interactions with different animals, which could specifically benefit from the inclusion of movement and sound in their presentation (47).

## Potential Benefits of Implementing Video-based Stimulus Preference Assessments for AAI

Preliminary research suggests that animals may be incorporated into SPA. A recent study by Protopopova et al. recently used SPA with children with intellectual and developmental disabilities, rewarding the achievement of academic tasks with the access to leisure items or a dog (48). The dog was identified as a strong reinforcer, more so than the leisure items. In this study, the animal was present in the room in front of the child during the preference assessment. This finding is encouraging and suggests that SPA is a feasible technique to identify preferred animals. When the choice is between one animal and inanimate items, the animal can easily be in the intervention room. But when trying to determine preference between multiple animals, the presentation of videos may be a useful alternative to preserve animal welfare and ensure feasibility.

A key characteristic of video-based preference assessments is their ability to feature more salient characteristics of the stimuli than pictorial, static formats. The incorporation of video assessments into the field of behavior analysis interventions has allowed researchers to broaden the utility of preference assessments into areas previously unavailable to incorporate choice in daily life, such as work preference and job selection (45, 49). This advancement has allowed individualized preference to be established with stimuli that are abstract, complex, and activity-based (47). This is especially relevant to AAI because the movement, sound, and interaction elements of AAI are important to display in stimuli that cannot be displayed through pictures. For example, while a static picture of a rabbit might depict physical characteristics of the animal, a video of a human interacting with the rabbit (e.g., brushing, stroking, or holding it) can include sounds that the animal might make as well as laughs, smiles, and giggles of the participant. Thus, the *interaction* can be captured rather than the animal alone. When using pictures, an individual’s repeated choice of a picture of a rabbit over a picture of a cat might be attributable to unknown characteristics of the picture’s contents. However, repeated choice of a video of stroking a rabbit over stroking a cat might parse out the particular interest of the participant’s desire to touch the animal rather than to simply view the animal.

Another primary advantage of using video-based preference assessments in AAI is that an individual can complete a session without needing to be directly exposed to animals. Preliminary research suggests that during preference assessments, obtaining contingent access to an activity after the choice is not necessary. For example, Clark et al. presented items via video to children with ASD in a paired-stimulus video format without providing access contingent on selection and found that even if the individual did not physically have access to the item, highly preferred stimuli did function as reinforcers (50). Brodhead et al. (38) extended the work of Clark et al. and found similar results when assessing preference for activities. Finally, Brodhead and Rispoli demonstrated that video assessments may also be used to accurately assess preference for novel stimuli (51). That is, this method may be used to assess preference for stimuli with which the individual has not yet interacted.

The above findings have important implications for feasibility because it seems unlikely that a clinician wishing to incorporate animal interactions into a therapy or intervention program would have access to a wide variety of animals at one time. Even in the case of larger therapy centers specialized in AAI, where the access to many sizes and species of animals is not a problem, presenting multiple animals at the same time may be logistically difficult and detrimental to animal welfare. Thus, it is more appropriate and plausible that an animal is introduced after the individual has completed a brief assessment and a preferred animal was identified. At that point, the activity or therapeutic intervention has been appropriately designed and maximizes the efficient use of both the animals’ and humans’ time.

Another potential benefit for implementing a preference assessment prior to designing an AAI involves avoiding unknown or unpredictable phobias. Animals can be a common fear among children, especially among low-functioning or non-verbal individuals. Though teachers and parents can report a known phobia, studies have shown that caregiver reports can often be unreliable (38, 39). As discomfort can be triggered by showing a participant a video of a known feared animal (52), a preference assessment is likely to identify a phobia. Doing so before a participant is presented with a live feared animal will help to avoid undue stress and anxiety in AAI participants.

## Considerations and Limitations in the Application of Video-based Stimulus Preference Assessments for AAI

Although video preference assessments might have advantages for use in AAI, their implementation raises several considerations. First, picture or video-based assessments are limited by the prerequisite of the individual’s ability to associate the animal seen on video with a live, tangible animal. This ability to associate an object seen on video and a tangible object is referred to as video-to-object and object-to-video correspondence. In previous research comparing two matched tangible and pictorial preference assessments, Clevenger and Graff found that only individuals who were capable of picture-to-object and object-to-picture matching skills had similar preference hierarchies (53). It is recommended that assessments using video or pictures be used only with participants who display 80% accuracy of video-to-object and object-to-video matching skills in a matching assessment test. This prescreening would limit the pool of participants for whom these assessments are applicable, and therefore may not be possible for all individuals with ASD.

Second, a limitation of the implementation of SPAs into the field of AAI and a critical area for research is the untested assumption that a highly preferred animal interaction presented in a video will translate to an effective in-person activity for the individual receiving the intervention. Although a plethora of research has investigated the predictive validity and reliability of reinforcers identified from SPAs of all modalities (37, 54), further research is needed to extrapolate conclusions drawn from previous literature to the novel use of SPAs for animal interactions.

## Implementing an SPA into the Design of AAI

The specifics of the protocol to incorporate SPA into AAI remain to be developed and assessed for feasibility and relevance. Practical elements to determine are the design of the SPA, the medium used to present the SPA, and the content and format of the videos. A brief multiple-stimulus paradigm with several video stimuli is the gold standard in the field of ABA and would be adapted to showing videos of human–animal interactions.

The content of the video should reflect the animal with which the participant may interact, in a context representative of the actual interaction that is offered. Depending on the goal of the SPA and the choice presented, the videos could represent animals from different species (e.g., a guinea pig or a cat), or different individual animals of the same species (e.g., two different dogs). The behaviors and the types of interactions with humans that are presented in the videos should be directed by the goals or nature of the intervention. For example, the videos could show the animals eating, being groomed, or playing with people. In addition, capturing the animals’ natural noises may increase the fidelity of the video to the animals and help participants make an informed choice.

The videos should be presented in a format such that participants can easily select or point to a preferred stimulus. In the field of ABA, computers and more recently handheld tablets have been used to identify preferred stimuli. An example of an effective design could incorporate an adaptive program that simultaneously presents multiple previews of videos of animal interactions and allows the participant to select a video to play in full screen for a few seconds. When back to the main screen, the participant can choose any other video to watch next. Each time a video has been played, it disappears from the main screen. When all animals have been selected, all of the videos are available from the main screen again for a second round of viewing and eventual preference selection.

Before any large-scale implementation, researchers should test any animal preference assessment method for predictive validity. A first step would be to compare the choice identified during the SPA before and after the participant has met the animals. If the same animal is preferred both before and after the participant has had a chance to interact with all the animals presented in the SPA, it is likely that the stimuli presented were representative. This would confirm the feasibility of identifying a preferred animal from a video.

If the predictive validity of an SPA for AAI is established, the next step will be to examine the clinical relevance of identifying a preferred animal during a therapeutic intervention or AAA. We hypothesize that preferred animals will yield more positive outcomes from AAI, as participants may feel a stronger sense of bonding or affinity with animals with which they chose. This assumption could be tested by comparing the outcomes of interventions preceded by an SPA or not, and within interventions preceded by an SPA, led with an animal either identified as preferred or non-preferred. Better outcomes from the intervention with the animal identified as preferred would then be a sign that the use of SPA offers a significant enhancement to AAI.

## Conclusion

Stimulus preference assessments have been widely implemented in the field of ABA in order to incorporate the desires of those with communicative difficulties to interventions. With heavily validated, tested, and refined methodology, SPAs may be a useful tool for the field of AAI. However, although SPAs have been widely used and empirically researched in the field of ABA, it is important to note that this is a currently unexplored and untested strategy for AAI. While some clinician guidelines for implementing AAI do suggest to control for the potentially confounding variables of pet ownership, animal aversion, and any allergies (55), there is not a best-practice guideline in place for determining a suitable AAI for those who are non-verbal or particularly low-functioning, such as those with severe ASD. Therefore, preference assessments may serve as a starting point for the field to become more systematic in incorporating different types of animals into research and practice.

In summary, we have proposed that integrating preference assessment methodology into AAI practices is a promising way to maximize positive outcomes for individuals who are severely disabled or experience communicative difficulty. Moreover, beyond simply assessing preference, it also may act as a way to rule out AAI as a possible treatment for an individual with ASD who is averse to animals or might not explicitly benefit from or exhibit positive emotions from an animal interaction. Further, behaviors that indicate aversion such as avoidance (56) or a lack of response (57) should be monitored during any sort of animal-focused SPA. This would serve to both avoid possible distress for the individual with ASD while both saving time and investment that would be involved during the AAI, and protecting animal welfare.

By incorporating individual preferences into AAI, clinicians will be able to better identify and avoid animals that a participant might be averse to and/or incorporate animals for which the individual might have a particular affinity. Ultimately, an ideal use of SPAs in the field of AAI could aid in increasing the efficiency and efficacy of the interventions that include animals, possibly by even determining its feasibility before starting. Additionally, we hope that the exploration of this practice will eventually allow for a broader application of incorporating animals into improving the mental health and well-being of those with ASD, who might have been restricted from AAI in the past because of their communicative limitations. Applications include the choice of a preferred animal prior to AAI, and the choice of activities during AAI. While the use of SPAs in identifying animals for therapeutic interventions is still exploratory, we believe that there are benefits to be gained from implementing this practice and encourage AAI researchers to consider the effects of incorporating preference and aversions into their research.

## Author Contributions

NG and KR are co-first authors; both wrote the body of the text and articulated the main ideas presented in this paper. MB provided extensive guidance and expertise in the area of applied behavior analysis. MO provided expertise in the area of animal-assisted intervention.

## Conflict of Interest Statement

The authors declare that the research was conducted in the absence of any commercial or financial relationships that could be construed as a potential conflict of interest.
